# Treatment challenges of sigmoid-shaped esophagus and severe achalasia

**DOI:** 10.1016/j.amsu.2020.11.077

**Published:** 2020-12-01

**Authors:** Ahmed Hammad, Vivian F. Lu, Dushyant Singh Dahiya, Asim Kichloo, Faiz Tuma

**Affiliations:** a Department of Surgery, Central Michigan University College of Medicine, Saginaw, MI, USA; b Department of General Surgery, Mansoura University, Egypt; c Department of Internal Medicine, Central Michigan University College of Medicine, Saginaw, MI, USA

**Keywords:** Achalasia, Sigmoid esophagus, Severe achalasia, Heller's myotomy with dor fundoplication, Esophagectomy

## Abstract

**Background:**

Achalasia is a chronic motility disorder which may require surgical interventions to effectively manage patients’ symptoms and improve functional status. In late stage achalasia, patients may present with sigmoid-shaped esophagus which complicates traditional treatment approaches for achalasia as the esophagus is massively dilated and dysfunctional with delicate tissue integrity. Severe Achalasia with sigmoid esophagus imposes significant challenge to surgeons and treating physicians. Various assessment modalities and treatment approaches have been tried. Surgical treatment continues to be controversial. Some have argued that a less aggressive approach similar to that in early Achalasia results in satisfactory outcomes. Others have argued a more aggressive approach of esophagectomy is necessary. We present a review of the challenges encountered in each approach with recommendation for selecting the right treatment for the individual cases.

**Conclusions:**

Different treatment options for sigmoid type achalasia are available with ongoing controversy among the options. Heller myotomy with Dor fundoplication can provide satisfactory symptoms improvement and treatment outcomes.

## Highlights

• Late stage achalasia may present with sigmoid-shaped esophagus.
• Sigmoid shaped esophagus imposes significant treatment challenges due to dilation and delicate tissue integrity.
• Surgical treatment continues to be controversial.
• Heller myotomy with Dor fundoplication can provide satisfactory symptoms improvement and treatment outcomes.

## 1 Introduction

Achalasia is a rare disease of the esophagus characterized by the inability of the lower esophageal sphincter (LES) to relax and the absence of esophageal peristalsis. The incidence and prevalence of achalasia is 1.63/100,000 and 10.82/100,000 respectively [1]. Recent studies have reported an increase in incidence over the years [2]. However, greater awareness and improved diagnosis of achalasia may account for the increase of incidence.

Patients with achalasia typically present with dysphagia for solids and liquids, regurgitation of undigested food, heartburn, and chest pain [3]. These clinical findings are considered in the Eckardt Symptom Score, a self-reported score used to evaluate the symptoms of achalasia and efficacy of achalasia treatment (Table 1) [4]. It has been reported that dysphagia occurs in over 90% of achalasia patients [5]. Dysphagia can be largely categorized as a motility disorder (achalasia, oropharyngeal dysfunction) or be mechanical in origin (esophageal stricture, malignancy, or extrinsic compression). Generally, symptoms from motility disorders occurs with both solids and liquids as the neuromuscular forces required to propel the bolus affects solids and liquids in a similar fashion. However, symptoms from mechanical disorders primarily occur with ingestion of solids and not liquids [6]. Given the nonspecific clinical presentation of achalasia, physicians may mistake symptoms of achalasia for gastroesophageal reflux disease (GERD), pseudoachalasia due to malignancy, motility disorders, and obstructions.

**Table 1:** Clinical scoring system for achalasia (eckardt symptom score).

Score	Weight Loss (kg)	Dysphagia	Retrosternal Pain	Regurgitation
0	None	None	None	None
1	<5	Occasional	Occasional	Occasional
2	5–10	Daily	Daily	Daily
3	>10	Each meal	Each meal	Each meal

The etiology of achalasia is largely unknown, and studies have suggested a multi-factorial cause [7]. It has been postulated that achalasia is linked to a viral or autoimmune inflammatory response, leading to selective degeneration of inhibitor neurons of the esophageal myenteric plexus [3]. However, the diagnosis of achalasia is very well established. Patients suspected of achalasia undergo diagnostic examinations such as esophagogastroduodenoscopy (EGD), barium swallow test, and chest CT [8]. Ultimately, a definitive diagnosis is made with manometry [9].

As achalasia progresses, dilation of the esophagus worsens and can resemble a sigmoidal shape. In late stage achalasia, patients present with a sigmoid esophagus which is defined as a dilation of the distal esophagus to more than 10 cm in diameter and/or one that takes a tortuous course through the chest towards the gastroenteric junction [10]. Treatment for late stage achalasia with severely dilated and sigmoidal shaped esophagus has been controversial. Some have argued that a laparoscopic Heller myotomy with Dor fundoplication, as used to treat early stage achalasia, have promising outcomes for late stage achalasia. Others have argued a more aggressive approach of esophagectomy [10]. This article presents a review of the challenges with severe achalasia and sigmoid esophagus, in terms of diagnosis, management, and outcomes.

### 1.1 Natural history and assessment of achalasia

Delayed diagnosis plays a large role in the development of end stage achalasia. It has been reported that the average delay between symptom onset and diagnosis of achalasia is 4–5 years [11]. Progressive dilation of the esophagus leads to a tortuous esophagus resembling a sigmoid shape and end stage achalasia. A barium swallow visualizes the esophagus anatomy including a classic “bird beak” appearance of the distal esophagus tapering and proximal esophageal dilation. In end stage achalasia, a barium swallow can identify severe proximal dilation resulting in a sigmoid-like appearance in the distal esophagus [12]. A New Japanese classification for esophagus was published in 2012 that further divided esophageal morphology into 3 categories: straight, sigmoid, advanced sigmoid based on its x-ray findings and degree of angulation [13].

An EGD allows visualizing the esophageal mucosa and lumen with and obtaining biopsies to evaluate for esophagitis, reflux disease, stenosis, and malignancy [14]. Its role in diagnosing achalasia, however, is somewhat limited. Only 41.1% patients with achalasia have endoscopic feature of esophageal dilation with tortuosity of large amount of food remnant [12]. Typical endoscopic findings suggestive of achalasia is a dilated esophagus with resistance to the passage of the endoscope through the gastro-esophageal junction and incomplete relaxation of the LES with air insufflations [15]. Patients with severe achalasia may have significant undigested food retained in the esophagus or require longer fasting periods prior to endoscopy.

Manometry is the gold standard for achalasia diagnosis. It can clearly delineate the lack of peristalsis and absent or incomplete LES relaxation [1]. The more advanced high-resolution manometry (HRM) provides a comprehensive description of the pressures that scores achalasia under the Chicago classification based on the pattern of contractility. In Type I, no pressures are recorded in the distal esophagus, Type II has panesophageal pressurizations, and Type III has spastic simultaneous contractions [16].

### 1.2 Stages of achalasia

Achalasia is sub-classified in 4 stages (Table 2): stage 0, esophageal width of 4 cm or less; stage I, width of between 4 and 6 cm; stage II, width of greater than 6 cm; stage III, or sigmoid esophagus representing the most advanced stage [15,17]. Sigmoid Achalasia is defined as marked dilation of the distal esophagus to more than 10 cm in diameter, having a tortuous course through the chest towards the gastro-esophageal junction (GEJ) and angulation of debris-filled esophageal lumen [18]. Progression of disease in the absence of any treatment leads to axis deviation [19].

**Table 2:** Stages of esophageal achalasia.

Stages	Description
stage 0	esophageal width of 4 cm or less
stage I	esophageal width of between 4 and 6 cm
stage II	esophageal width of greater than 6 cm
stage III	marked dilation of the distal esophagus >10 cm in diameter, tortuous course, angulation ± axis deviation

The dilation of the esophageal body into a massively dilated sigmoid esophagus (stage III) is slow and gradual with long-standing achalasia in patients with phenotypes I or II (Chicago classification), usually in the elderly [20]. In this stage, patients have aggravated symptoms and significant, nutritional and pulmonary complications due to severe food stasis and regurgitation [21]. It is possible that the inflammatory changes noticed in the muscle layers may be an extension from the adjacent inflammatory process in the Auerbach plexus [22].

The severity of the disease is staged by evaluating the dilatation degree of esophageal body in centimeters by measuring the maximum esophageal width from standard posteroanterior projection esophagograms [23]. Manometric findings of concurrent esophageal body contractions along with high LES basal pressure and inadequate relaxation of LES to wet swallow are diagnostic [24]. Chest radiography can show a widened mediastinum, a mediastinal air-fluid level, or absence of a gastric air bubble [21]. Computed tomography (CT) offers ancillary findings of grossly dilated fluid-filled esophagus, tracheobronchial tree compression and the frequently seen lung changes as sequela of aspiration like consolidation, fibrotic patch, ground glass or nodular opacities [25]. A diameter of >6 cm and axis deviation are regarded to represent a sigmoid esophagus, the distal esophagus is kinked toward the left, outside of the esophageal axis [26].

Stasis of ingested food results in worsening dysphagia, frequent aspiration, weight loss, and cachexia [18,27]. Respiratory symptoms can be attributed to chronic micro-aspiration of the retained food residue in the lower end of the esophagus and compression of the tracheobronchial tree by the dilated esophagus [28].

Chronic inflammatory irritation by retained food may cause esophageal squamous epithelial dysplasia [29]. The prognosis of achalasia-associated carcinoma is poor, as symptoms are usually hidden by severe dysphagia of long-term achalasia. Thus, they often diagnosed in the advanced stages, and the incidence of early carcinoma was reported only 9.1% [30]. Therefore, endoscopic surveillance using iodine staining has been recommended in such patients [31] Radiographically, most of the patients lose peristalsis, and distal esophageal stricture, retention of barium and food material in the esophagus is seen. Endoscopically, esophageal food stasis and chronic hyperplastic esophagitis together with iodine non-stained lesions [32].

### 1.3 Treatment options

Symptomatic relief can be provided by means of surgery [15]. However, peri-operative respiratory symptoms including chronic/nocturnal cough, dyspnea and acute respiratory symptoms like choking spells and asphyxia are occasionally encountered. The structural alterations of the esophagus become irreversible in end-stage sigmoid esophagus and treatments such as mechanical pneumatic dilation, botulinum toxin injections, and myotomy are rendered less effective in the long term, often leading to need for re-treatment [33].

Treatment of patients with sigmoid-type achalasia has been controversial. Some surgeons recommend myotomy as the initial treatment and reserve esophageal resection only for patients with persistent symptoms [22] since esophageal replacement with esophagectomy is more technically demanding [34,35]. Others recommend esophagectomy as the first choice, presuming that marked esophageal dilation and redundancy hinder the possibility of improving emptying by means of a simple myotomy [34,[36], [37], [38]]. However, esophagectomy is also associated with complications ranging from mild nocturnal regurgitation, mild dumping symptoms (cramps and diarrhea) to laryngeal nerve injury, tracheal tear, bleeding, chylothorax, pleural effusion, anastomotic leakage, cervical fistula, reflux esophagitis and Barrett's esophagus in the esophageal stump [39]. Moreover, esophagectomy bears a mortality rate of about 3% for treating sigmoid esophagus even by an experienced surgeon [26,38]. Furthermore, anastomotic dilatation for the relief of post-surgical and/or recurrent dysphagia due to stenosis of the cervical esophagogastrostomy [40] may be required in 38.5–50% of patients and dumping symptoms have been reported in 4–19% of patients [41].

### 1.4 Heller myotomy with Dor fundoplication

Successful treatment of sigmoid-shaped esophagus with laparoscopic Heller myotomy (LHM) has been demonstrated by multiple studies, supporting LHM as a primary surgical treatment for sigmoid achalasia [42,43]. To prevent postoperative gastroesophageal reflux (GER) after LHM, addition of an anti-reflux procedure like Toupet, Dor fundoplication or angle accentuation were established. LHM with Dor's fundoplication is more effective and safer procedure for avoiding GER, dysphagia, mucosal perforation, and/or pseudo-diverticulum [44]. The anterior 180° Dor has emerged as the wrap of choice as a standard (LHM + Dor procedure) [[45], [46], [47], [48]]. Mega-esophagus or sigmoid-shaped esophagus is predictive of a less favorable outcome in patients treated with a Heller myotomy [49]. The operative procedure is modified to correct the esophageal axis deviation. Circumferential mobilization of the esophagus and extensive mediastinal dissection to straighten the horizontal lower end of esophagus adds additional time. Further, such patients usually have significant *peri*-esophageal inflammation due to long-standing retention of food with subsequent oesophagitis, and the dissection becomes more challenging. This may be also secondary to prior interventions such as dilatations or prior botulinum toxin injections [50]. It may be noted that the median duration of dysphagia in patients with sigmoid esophagus is 55 months, much longer than the median duration of 36 months seen in patients with non-sigmoid achalasia [51].

Sweet et al. [46], reported that Heller myotomy with anterior Dor fundoplication was highly successful even mega or sigmoid esophagus. Swallowing improved in 90% of patients with acceptable palliation of the disabling dysphagia, regurgitation, chest pain, heartburn, retrosternal pain, aspiration, weight loss and recurrent lower respiratory infections in more than 80% of patients. Patients also saw significant improvements in quality of life [23,52,53]. Faccani et al. [21], reported the pull-down technique or the verticalization of the esophageal axis to improve the clinical outcomes of LHM + Dor for sigmoid achalasia (diameter >6 cm)^,^ particularly when kinked to the left and outside of the esophageal axis. The phreno-esophageal membrane is divided anteriorly, and the anterior wall of the stomach is pulled down. The gastro-esophageal junction and the lower mediastinal esophagus are fully mobilized for at least 6 cm. Prior to performing the Heller–Dor procedure, two or more U intramuscular stitches are applied at the level of the esophageal curling to pull down and rotate the side of the GEJ with a tie of the sutures [21,54].

Some patients are offered esophagectomy in consideration of the size of their esophagus (>6 cm), their younger age <55 years, having recurrent infection secondary to esophageal stasis or severe mucosal inflammation and moderate to severe dysplasia [55]. Esophagectomy may be offered after Heller myotomy for cancer implanted in their mega-esophagus after one or more myotomies and for postsurgical scar stenosis of the GEJ as well as those with failed a redo myotomy if symptoms are severe and quality of life is impacted [32]. In the previous studies, the mean interval from onset of dysphagia in achalasia patients to diagnosis of esophageal squamous cell carcinoma was 17–21.5 years [28]. In the myectomy cases, the carcinoma was diagnosed after a mean period of 17 years of post-operative follow-up [56]. The frequency of cancer arising in sigmoid achalasia is not definitively defined; however, the data collected to date may not force surgeons to esophagectomy in the absence of dysplasia [52]. Only 8.3% of patients with the most severely dilated esophagus did require an esophagectomy after LHM + Dor for 6 cm sigmoid-shape achalasia [26,57]. Minimally invasive esophagectomy offers similar symptom relief but was linked with a longer hospital stay relative to LHM [22,57].

### 1.5 Esophagectomy

When esophagectomy is carried out for achalasia, the esophagus is markedly dilated, and great caution must be exerted by the anesthesiologists upon induction to prevent aspiration [49]. The esophageal wall is generally thickened and is supplied by engorged collateral blood vessels, requiring extra care for hemostasis. Esophageal body dilation may extend up to the cervical level, and care must be taken to prevent injury to the recurrent laryngeal nerves, particularly on the left. If a fundoplication is present and the stomach is being considered as the esophageal replacement conduit, the fundic wrap should be undone [50]. Generally, the stomach will have adequate length and vascularity to allow a cervical anastomosis, if desired. Exceptionally, prior operations involving the greater curvature of the stomach may have disrupted the right gastroepiploic artery, critical to the blood supply of a planned gastric pull-up, making a planned colon interposition necessary. Preoperative evaluation and preparation of the colon are advisable in case it is needed as an esophageal substitute [28].

In patients with end-stage achalasia, the esophagus may be relatively difficult to dissect owing to periesophageal adhesions secondary to transmural fibrosis, prior operation, or esophageal body dilation with neovascularization. Thus, a transthoracic route, either open or minimally invasive, would be preferred in terms of safer dissection under direct visualization, but a *trans*-hiatal route, with an experienced surgeon, has much less complicated postoperative behavior, minimizing respiratory complications [21]. Anastomosis must be performed in the neck, with the esophageal replacement conduit passing through the esophageal tunnel. Pyloroplasty helps to facilitate gastric emptying [52].

Other surgical options that have been used in the management of massively dilated esophagus include laparoscopic esophagogastrostomy with posterior hemi-fundoplication, distal esophagectomy with antrectomy and Roux-en-Y diversion, esophagectomy with gastric, colon or jejunal interposition, esophagocardioplasty, vagotomy-antrectomy, and Roux-en-Y gastrojejunostomy (Serra Doria operation), Y-cardioplasty truncal vagotomy for selected cases, partial distal gastrectomy, subtotal esophagectomy, Vagal-sparing esophagectomy and Vertical esophagectomy + myotomy [58,59].

### 1.6 Per-oral endoscopic myomectomy in sigmoid-type achalasia

Per-oral endoscopic myomectomy (POEM), is a novel less invasive approach that may offer another option. Complication rates are low, symptom control is excellent and systematic reviews of published series show similar or slightly superior dysphagia control with POEM than with LHM [26,60]. Advantages of POEM are the possibility to perform a long-myotomy (of the entire length of the esophagus if necessary) and the relatively free choice of the localization of the myotomy (anterior/posterior POEM) [61]. The disadvantage is the increased postoperative gastroesophageal reflux after POEM; however, this sequel is controlled with PPI in most cases, or a future laparoscopic fundoplication, if necessary [62].

The morphological changes of sigmoid-type achalasia make the POEM operation much harder and time-consuming than non-sigmoid cases [63]. Severe sigmoid esophagus can markedly increase the technical difficulty of POEM procedure. Patients with severe esophageal stasis may have inflammation and fibrosis of the submucosa, hindering submucosal tunneling; severe angulations also make the direction of submucosal tunneling very puzzling, and should only be performed by highly experienced endoscopists [64]. Thus, there is an increased risk of mucosal perforation due to decreased submucosal cushioning or tunneling into the intermuscular space, leaving circular muscle adherent to the mucosal side uncut [65]. If there, esophageal candidiasis should be eradicated with an antifungal agent before performing POEM [65]. However, use of POEM in advanced staged patients has been reported with good feasibility and short-term success [61,66].

In advanced sigmoid achalasia, the esophagus can be tortuous, both distally and proximally. Because of the severe dilation, the spine and trachea indentations on the esophagus are also exaggerated, which can make maneuvering the endoscope more difficult, particularly in the proximal esophagus. In advanced disease, the thoracic esophagus tends to deviate to the right, only to shift back over to the midline at the GEJ [20]. This phenomenon should be taken into account because it can render luminal landmarks less reliable [25]. In addition, while creating the submucosal tunnel, acute angulations in the esophageal body may be mistaken for the GEJ [24]. If the operator is not experienced and fails to correctly advance into the stomach because of misinterpreting an angulation for the GEJ, the result will be a failed POEM procedure [60].

Caution is required during the mucosal incision due to decreased vertical elevation after the initial submucoal injection from fibrosis. If the mucosal incision is performed hastily, a full-thickness incision into the mediastinum may result, with subsequent dreaded mediastinitis [25]. Therefore, every millimeter of movement in POEM must be calculated and meticulously controlled. With advanced sigmoid achalasia there is also neovascularization, resulting in increased density and caliber of vessels encountered during the procedure, leading to an increased risk of intraprocedural bleeding [67,68]. In addition, the mucosa can be significantly thickened, leading to challenging closure of the mucosal incision [20]. In fact, most cases that have reported the use of alternative closure methods, such as fully covered stents, over-the-scope clips, and end loops with clips, have involved advanced sigmoid achalasia. Cases of advanced sigmoid achalasia poses many challenges and complications and should only be performed by highly trained operators [69].

Sigmoid-type esophagus was another independent risk factor for the occurrence of gas-related complications in POEM [65,66]. Due to esophageal twisting, it is difficult to create a submucosal tunnel in patients with sigmoid type esophagus. And the curvature interferes with gas discharging out of the tunnel, which might form a state of high pressure within the tunnel to cause such complications as subcutaneous emphysema, pneumothorax and pneumoperitoneum [70]. During tunneling, bypassing the sites of the most serious esophageal twisting or invagination is essential to decrease the difficulty of tunnel creation and prevent tunnel obstruction. The tunnel width of a sigmoid-type esophagus should be wider than that of a non-sigmoid-type or more half of esophageal circumference [61,65,66].

## 2 Conclusions

Severe achalasia with sigmoid esophagus poses a significant challenge in management. The treatment options available for sever achalasia with a sigmoid esophagus are highly controversial. Literature has demonstrated successful treatment of sigmoid-shaped esophagus with laparoscopic Heller myotomy (LHM) by multiple studies, supporting LHM as a primary surgical treatment for sigmoid achalasia. It is considered safer and effective procedure to avoid gastroesophageal reflux, dysphagia, mucosal perforation, and/or pseudo-diverticulum. However, management of severe achalasia should always be individualized to the patient and requires a thorough risk-benefit assessment. Patient education and extensive discussions of the expectation from the procedure is essential prior to the procedure. Additionally, like reported in literature, the authors prefer performing Heller myotomy with Dor fundoplication for patients with severe achalasia and sigmoid esophagus as it provides satisfactory symptom improvement and treatment outcomes.

### 2.1 Provenance and peer review

Not commissioned, externally peer reviewed

## Conflicts of Interest and Source of Funding

The authors report no conflicts of interest and no funding

## Author Contributions

**Ahmed Hammad, MD, PhD:** Writing the draft, reviewing/editing. **Vivian F. Lu, BS:** Writing the draft, data collection, review/editing. **Dushant Singh Dahiya MD:** Writing the draft, review/editing. **Asim Kichloo MD;** revising/editing, visualization, supervision. **Faiz Tuma MD, MME, MDE, EdS, FACS, FRCSC:** Conceptualization, writing/review/editing, visualization, supervision.
